# Microbial treatment of alcoholic liver disease: A systematic review and meta-analysis

**DOI:** 10.3389/fnut.2022.1054265

**Published:** 2022-11-21

**Authors:** Qinjian Wang, Jiangmin Shi, Min Zhao, Gaoyi Ruan, Zebin Dai, Yilang Xue, Dibang Shi, Changlong Xu, Ouyue Yu, Fangyan Wang, Zhanxiong Xue

**Affiliations:** ^1^Department of Gastroenterology, The Second Affiliated Hospital and Yuying Children’s Hospital of Wenzhou Medical University, Wenzhou, China; ^2^Department of Pathophysiology, School of Basic Medicine Science, Wenzhou Medical University, Wenzhou, China

**Keywords:** alcoholic liver disease, microbial agents, probiotics, prebiotics, gut-liver axis, meta-analysis

## Abstract

**Background and aims:**

Alcoholic liver disease (ALD) is characterized by impaired liver function due to chronic alcohol consumption, even fatal in severe cases. We performed a meta-analysis to determine whether microbial agents have therapeutic potential for ALD and elucidate the underlying mechanisms.

**Methods and results:**

Forty-one studies were eligible for this meta-analysis after searching the PubMed, Cochrane, and Embase databases. The combined analysis showed that microbial therapy significantly decreased hepatic enzymatic parameters, including alanine transaminase [standardized mean difference (SMD): –2.70, 95% confidence interval (CI): –3.33 to –2.07], aspartate aminotransferase (SMD: –3.37, 95% CI: –4.25 to –2.49), γ-glutamyl transpeptidase (SMD: -2.07, 95% CI: –3.01 to –1.12), and alkaline phosphatase (SMD: –2.12, 95% CI: –3.32 to –0.92). Microbial agents endotoxin to enter the portal circulation and increasing reduced total cholesterol (SMD = -2.75, 95%CI -4.03 to -1.46) and triglycerides (SMD = –2.64, 95% CI: –3.22 to –2.06). Microbial agents increased amounts of the beneficial flora *Lactobacillus* (SMD: 4.40, 95% CI: 0.97–7.84) and *Bifidobacteria* (SMD: 3.84, 95% CI: 0.22–7.45), *Bacteroidetes* (SMD: 2.51, 95% CI: 0.29–4.72) and decreased harmful *Proteobacteria* (SMD: –4.18, 95% CI: –6.60 to –1.77), protecting the integrity of the intestinal epithelium and relieving endotoxin (SMD: –2.70, 95% CI: -3.52 to –2.17) into the portal vein, thereby reducing the production of inflammatory factors such as tumor necrosis factor-α (SMD: –3.35, 95% CI: –4.31 to –2.38), interleukin-6 (SMD: –4.28, 95% CI: –6.13 to –2.43), and interleukin-1β (SMD: –4.28, 95% CI: –6.37 to –2.19). Oxidative stress was also relieved, as evidenced by decreased malondialdehyde levels (SMD: –4.70, 95% CI: –6.21 to –3.20). Superoxide dismutase (SMD: 2.65, 95% CI: 2.16–3.15) and glutathione levels (SMD: 3.80, 95% CI: 0.95–6.66) were elevated.

**Conclusion:**

Microbial agents can reverse dysbiosis in ALD, thus significantly interfering with lipid metabolism, relieving inflammatory response and inhibiting oxidative stress to improve liver function.

## Introduction

Alcoholic liver disease (ALD) is a spectrum of diseases, including steatohepatitis, alcoholic hepatitis, cirrhosis, and associated complications, which in severe cases can progress to liver failure and lead to multi-system dysfunction (1). The dynamics of ALD involve interactions between the direct toxicity of alcohol and its metabolites, oxidative stress, inflammatory cascades, and other complex alcohol-related consequences (2). The optimal management strategy for ALD is still debated due to the limited efficacy of current treatments (3).

Several lines of evidence suggested a link between intestinal flora and liver diseases. The overgrowth of gram-negative bacteria and subsequently elevated gut-derived endotoxin were found in patients with ALD, participating in the pathogenesis of ALD (4). Chronic heavy alcohol consumption damages the intestinal epithelial barrier. Consequently, it increases intestinal permeability, allowing intestinal endotoxin to enter the portal circulation and increasing the production of pro-inflammatory cytokine IL-1β, TNF-α, and IL-6 through the activated toll-like receptor (TLR) 4-nuclear factor kappa B (NF-κ B) pathway (5).

Probiotics are living microorganisms that offer health benefits to the host the host health benefits, whereas prebiotics is non-digestible food ingredients that selectively stimulate the growth or activity of probiotics (6, 7). *Lactobacillus rhamnoses GG* helped prevent chronic alcohol exposure-induced hepatic steatosis by increasing hepatic AMPK phosphorylation and Bax-regulated apoptosis (8). In a mouse model of ALD, the probiotic Akkermansia muciniphilak reinforces the gut vascular barrier (GVB) and thus protects against alcohol-induced liver damage (9). Most probiotics targeting ALD attenuate the barrier disruption caused by ethanol exposure (as seen by a reduction in intestinal leakiness) and restore tight junction protein expression as well as the thickness of the mucus layer (10). *Bifidobacterium bifidum* and *Lactobacillus plantarum 8PA3* restore the normal intestinal flora in ALD, significantly enhancing liver function by mitigating liver-specific bio-enzymatic values (11). Prebiotic fructo-oligosaccharides increased the abundance of beneficial bacteria such as *Lactobacillus* and *Bifidobacterium* and improved alcoholic steatohepatitis (12). Pectin, as a prebiotic, restored intestinal homeostasis in mice with ALD, increasing the number of cupped cells and the expression of defensins Reg3β and Reg3γ (13). One study found that treatment by fecal transplantation from prebiotic (pectin)-fed mice prevented ALD (14).

Nevertheless, due to the diversity of microbial agents, the therapeutic effects of microbes on ALD have not been comprehensively described. In the present study, we collected data from published clinical research and preclinical studies and systematically assessed serum biochemical parameters, serum inflammatory parameters, and blood lipids to determine the effectiveness of microbial agents in ALD treatment. We also screened studies foroxidative stress parameters and intestinal barrier function to elucidate the underlying mechanisms.

## Materials and methods

### Literature search

Meta-analysis was conducted according to the Preferred Reporting Items for Systematic Reviews and Meta-Analysis (PRISMA) guidelines (15). PubMed, the Cochrane Library, and Embase were searched for studies in English up to November 2021. The search strategy was devised using medical subject headings and synonyms, as follows: (“prebiotics” OR “yogurt” OR “inulin” OR “oligosaccharide” OR “galactose oligosaccharide” OR “fructose oligosaccharide” OR “probiotics” OR “*Lactobacillus*” OR “*Bifidobacterium*” OR “*Enterococcus*” OR “*Streptococcus*” OR “*Saccharomyces*” AND disease “liver injury” OR “alcohol-induced liver injury” OR “Alcoholic liver disease”). After filtering the titles, abstracts, full texts and eliminating duplicates, appropriate studies were retained if they matched the inclusion criteria. Two reviewers carried out these screenings independently, and disagreements were resolved by discussion and consensus.

### Study selection

The literature included basic animal experiments and clinical trials. Animal experiments followed the PICOS principles (i.e., participants, interventions, comparisons, outcomes, and study design). Participants (P): Animal models of ALD, usually shaped by feeding with large amounts of alcohol. Intervention (I): The intervention group received microbial preparations, including probiotics and prebiotics. Comparison (C): the comparison group uses no microbial agents. Outcome (O): outcome indicators include several key components: (1) liver enzymes, alkaline phosphatase, γ-glutamyl transpeptidase (GGT); (2) blood lipids like triglyceride (TG), total cholesterol (TC); (3) inflammatory indicators such as TNF-α, IL-6, IL-10, endotoxin, malondialdehyde (MDA), superoxide dismutase (SOD). Study design (S): randomized controlled studies. The inclusion criteria for clinical trials were similar to those for animal trials Cohort studies, case-control studies, and cross-sectional studies were excluded during the selection process.

### Quality evaluation and data extraction

We included preclinical and clinical studies. For preclinical studies, methodological quality was assessed according to the Systematic Risk of Bias Evaluation Center for Laboratory Animal Experiments (SYRCLE) tool (16). Clinical studies were assessed using the Cochrane Risk Assessment Scale in the Cochrane Handbook. All experimental data were extracted independently and cross-checked by two authors. Graphical data were generated using Get Data software. The following information was extracted: (1) first author and publication year; (2) participant characteristics. It should be noted that, for animal experiments, we recorded animal species, modeling approach, and sample size. We recorded the age, the number of participants, and nationality for clinical experiments. (3) route of administration, dosage, and duration of treatment; (4) outcome variables. Discrepant opinions were resolved through third-party discussions.

### Statistical analysis

The 95% confidence interval (CI) and standard deviation of the combined mean difference were used to determine differences in continuous variables. We also used the Cochran Q-test and I^2^ statistic (*I*^2^ < 25%, low heterogeneity; 25–50%, moderate heterogeneity; *I*^2^ > 50%, high heterogeneity). Because animal studies and clinical trials are exploratory, a random effects model was used. A statistically significant difference was defined as *p* < 0.05. Seventeen indicators were analyzed in subgroups to explore the sources of heterogeneity based on the type of microbes, strains, animal models, and modeling approaches. A meta-regression was conducted to determine potential heterogeneity origins. A sensitivity analysis was conducted to identify studies that significantly influenced the results by eliminating them one by one. Publication bias was estimated quantitatively using Egger’s test. Contour-enhanced funnel plots obtained using the trim-and-fill method help to distinguish asymmetries caused by publication bias or heterogeneity, among other factors (17). If the missing study is in a non-significant region, the asymmetry is attributable to publication bias. Alternatively, the observed asymmetry could be attributed to factors other than publication bias. The statistical analysis was performed using R and R Studio software.

## Results

### Identification of relevant studies

**FIGURE 1:**
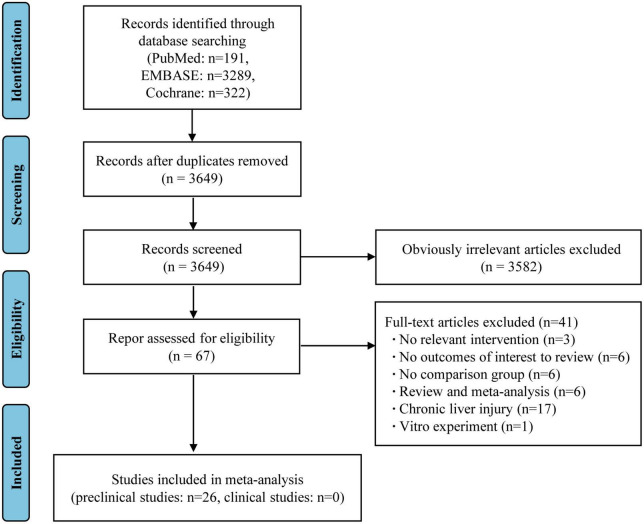
PRISMA flow diagram for included prospective cohort studies.

The flow diagram of this meta-analysis is displayed in Figure 1. A total of 9,858 records were initially obtained from the three databases, of which 3,484 were removed due to duplication. The initial screening of titles and abstracts yielded 87 articles after excluding 6,287 studies. A further 41 articles were rejected based on a detailed full-text evaluation. There were 41 studies (including 37 animal studies and four clinical studies) that met the inclusion criteria after a thorough screening of the full text (4, 11, 18–56).

### Study characteristics and quality assessment

Tables 1, 2 display the characteristics of the 41 studies. All animal experiments were carried out using rodent models, mainly C57BL/6N mice and Wistar rats, the microbial agents in the intervention group were primarily probiotics (mostly *Lactobacillus*), and placebos were usually used in the control groups. In the clinical studies, subjects were patients of various nationalities with alcoholic hepatitis and cirrhosis caused by chronic heavy alcohol consumption.

**TABLE 1:** Characteristics of included studies investigating the effects of probiotics and prebiotics on animal ALD.

References	Animal	Sample size	Modeling methods	Dosage	Route and duration	Comparison	Outcome indicators
Page et al. (15)	Adult Wistar rat’s female, 180–200 g	25	Orally treated with alcohol for 5 days	Probiotics: 14 × 10^10^ml^–1^ of *Lactobacillus acidophilus* 2 Bifidobacterium longum	Orally, for 2 5 days	Normal saline	AST↓,ALT↓ and Endotoxin↓
Hooijmans et al. (16)	Male C57BL/6N mice 7-week-old	24	Orally treated with alcohol for 5 weeks	Probiotics: 500 mg/kg of heat-killedL.brevis8803	Orally, for 5 weeks	Distilled water	AST↓,ALT↓,TG↓,TC↓ and TNF-α↓
Qing and Wang (18)	Male C57BL/6N mice	24	Orally treated with alcohol for 5 weeks	Probiotics: *Lactobacillus rhamnosus* GG (1 × 10^9^CFU/mouse per day)	Orally, for 2 weeks	Isocaloric maltose-dextrin	TG↓,ALT↓,Endotoxin↓,
Segawa et al. (19)	Male C57BL/6N mice 8-week-old	20	Orally Lieber-DeCarli liquid diet for 2 weeks and 5% (v/v) alcohol diet for weeks	Probiotics: *Lactobacillus acidophilus* (1 × 10^9^CFU/mouse per day)	Orally, for 2 weeks	Isocaloric maltose-dextrin	TG↓,ALT↓,LPS↓,and microbial transform
Stadlbauer et al. (20)	Female Wistar rats (200–250 g)	60	Oral gavage 10 g/kg/day of 35% (v/v) ethanol orally for 2weeks. 14 g/kg/day for next 10 weeks	Probiotics: 1 *L. plantarum* (10^10^cfu/mL). 2 AL-CA *L. plantarumbeads* (Equivalent to 10^10^cfu/mL)	Oral gavage,for 10 weeks	Distilled water	AST↓,ALT↓,ALP↓,Endotoxin↓ and TNF-α↓
Wang et al. (21)	Male mice (20 ± 2 g)	50	Orally gavage 6.25 mL of ethanol/kg per day for 5 weeks	Probiotics: *Lactobacillus* Whey fermented liquid	Oral gavage,for 5 weeks	Distilled water	AST↓,ALT↓,TG↓,GSH↑,SOD,and MDA↓
Bull-Otterson et al. (22)	Male C57BL/6N mice 8-week-old	20	Intragastric alcohol diets for 3 weeks	Prebiotics: 1. Unsaturated fatty acid; 2. Saturated fatty acid	Intragastric, for 3 weeks	Isocaloric diet	TG↓,ALT↓and microbial transform
Arora et al. (23)	Male Wistar rats 8-week-old	32	Orally treated with ethanol liquid diet for 12 weeks	Probiotics: Symbiotic supplementation	Orally, for 12 weeks	Normal liquid diet	ALT↓,TG↓,AST↓,TNF-α↓,IL-1β↓,Endotoxin↓ and microbial transform
Chen et al. (25)	Male C57BL/6 mice 8-week-old	100	Intra-gastric ethanol (5 g/kg/day twice/week for 9 weeks	Probiotics: *Lactobacillus rhamnosus* R0011 and acidophilus R0052 Prebiotics: KRG (Korea red ginseng); urushiol (*Rhus verniciflua* Stokes)	Intra-gastric,for last 2 weeks (1 mg/mL/day).	Normal chow diet	ALT↓,TNF-α↓,IL-1β↓
Hong et al. (28)	Male C57BL/6 mice 8–10-week-old	16–40	Orally treated with alcohol for 4 weeks	Probiotics: LGGs at a dose equivalent to 10^9^CFU/day/mouse	Orally, for 12 weeks	Isocaloric maltose dextrin	TNF-α↓,Endotoxin↓,
Chiu et al. (26)	Male Kunming mice (19 ± 1 g)	60	Orally treated with alcohol for 3 months	Probiotics: 1. *L. rhamnosus* CCFM1107; 2.LGG; 3. *L. plantarum* CCFM1112	Orally, for 3 months	Skimmed milk	AST↓,ALT↓,TG↓,TC↓,GGT↓,GSH↑,SOD↑,MDA↓and microbial transform
Han et al. (27)	Male C57BL/6N mice	16–40	Orally treated with 5% alcohol for 4 weeks	Probiotics: LGGs at a dose at equivalent to 10^9^CFU/day/mouse	Orally, for 4 weeks	Isocaloric maltose dextrin	AST↓,ALT↓,TG↓
Tian et al. (29)	Female 12-month-old mice	48	Orally treated with alcohol for 12 weeks	Probiotics: *Lactobacillus fermentum*	Orally, for 12 weeks	Normal chow diet	AST↓,ALT↓
Zhang et al. (30)	Male C57BL/6 mice 10 weeks of age	18	Orally treated with Liber-DeCarli diet containing 5% EtOH (w/v) for 10 days, and a bolus of EtOH (5 g/kg) was gavaged	Probiotics: *Lactobacillus rhamnosus* GG	Orally, for 10 days	Isocaloric diet	AST↓,ALT↓
Zhao et al. (31)	Male Spraque-Dawley 6-week-old	45	Orally gavaged with a single dose of alcohol	Probiotics: 1. *Lactobacillus salivarius*; 2. *Lactobacillus johnsonii*	Orally gavaged, for 10 days	Gavaged with normal saline.	ALT↓,AST↓,GGT↓,MDA↓,TG↓and TC↓
Barone et al. (32)	Male albino Wistar rats	36	Orally treated with30% ethanol (equivalent to 6 g/kg b.w. p.o) for 60 days	Prebiotics: Zingerone in different concentrations	Orally, for 60 days	Isocaloric glucose and dimethyl sulfoxide (DMSO)	AST↓,ALT↓, GGT↓and ALP↓
Chen et al. (33)	Female wistar rats (200–250 g)	60	Orally gavaged with alcohol	Probiotics: *Lactobacillus plantarum* MTCC 2621	Orally gavaged, for 8 weeks	Distilled water	ALT↓,AST↓,ALP↓,TNF-α↓
Chuang et al. (34)	Male ICR mice (21–23 g, 7 weeks old)	56	Orally gavaged with alcohol	Probiotics: 1.*Lactobacillus plantarum* LC27; 2. *Bifidobacterium longum* LC67; 3.LC27 + LC67	Orally gavaged, for 16 days	Vehicle (1% dextros)	ALT↓,AST↓,TC↓,TG↓,TNF-α↓,MDA↓ and microbial transform
Mani et al. (35)	Female C57BL/6 mice 8–10-week-old	48	Orally treated Lieber-DeCarli liquid	Probiotics: *Lactobacillus plantarum*	Orally, for 4 weeks	Isocaloric maltodextrin	AST↓,ALT↓,TNF-a↓ and TG↓
Rishi et al. (36)	Male C57BL/6J mice (22∼25 g)	48	Orally treated with alcohol for 6 weeks	Probiotics: *Lactobacillus plantarum*	Orally, for 6 weeks	Normal saline	ALT↓,AST↓,TC↓,TG↓,TNF-α↓,IL-1β↓,GSH↑,SOD,,Endotoxin↓,ZO-1↑,MDA↓ and microbial transform
Kim et al. (37)	C57BL/6 mice (8–12 weeks)	33	Orally treated with Lieber-DeCarli liquid with alcohol for 15 days	Prebiotics: Indole-3-acetic acid (IAA)	Orally gavaged, for 16 days	Normal saline	ALT↓,TG↓
Shukla et al. (38)	Sprague-Dawley rats 8-week-old	60	Orally gavaged normal chow diet and intragastric ethanol	Probiotics: 1. 1 ml/kg/day Golden Bifid 2. 1 ml/kg/day Medilac-S^®^ suspension 3. Golden Bifido suspension + glutamine	Orally gavaged, for 8 weeks	Chow diet and 1ml/kg/day saline	BW↑,AST↓,ALT↓, TG↓,TNF-α↓,Endotoxin↓ and microbial transform
Fang et al. (39)	Male Sprague-Dawley rats	30	Orally gavaged with 50% alcohol at 4 g/kg BW daily.	Probiotics: *Lactococcus chungangensis*	Orally gavaged, for 8 weeks	Phosphate buffer saline	ALT↓,AST↓,ALP↓,TC↓,TG↓,TNF-α↓,IL-1β↓and SOD↑
Hendrikx et al. (40)	Female C57BL/6J mice	60	Orally treated with Lieber-DeCarli liquid with alcohol for 6 weeks	Prebiotics: Inulin	Orally, for 6 weeks	Lieber-DeCarli liquid	BW↑,AST↓,ALT↓, IL-6↓,IL-10↓,TNF-α↓,Endotoxin↓ and microbial transform
Huang et al. (41)	Male Kunming mice 6-week old	60	Orally treated with 50% alcohol (v/v) at the concentration of 0.1 mL/10 g per day.	Probiotics: 1. *Lactobacillus plantarum* HFY 05; 2. *Lactobacillus delbrueckii* subsp. Bulgaricus	Orally, for 8 weeks	Normal saline	ALT↓,AST↓,ALP↓,TNF-α↓,SOD↑,MDA↓,IL-6↓and microbial transform
Yang et al. (43)	Male C57BL/6 mice	48	Orally treated with10% alcohol plus HFD	Probiotics: *Bifidobacterium longum*	Orally, for 6 weeks	Normal diet	ALT↓,AST↓,TC↓,TG↓,GGT↓,TNF-α↓,IL-1β↓,SOD↑,MDA↓ and IL-6↓
Yi et al. (44)	Male C57BL/6 mice	40	Orally treated with alcohol for 8 weeks	Probiotics: *Lactobacillus fermentum*	Orally, for 8 weeks	Isocaloric maltose dextrin	ALT↓,AST↓,TC↓,TG↓,Endotoxin↓,SOD,,MDA↓,IL-6↓,TNF-α↓and GSH,
Jiang et al. (45)	Male C57BL/6N mice 8-week-old	80	Orally gavaged ethanol, 5 g/kg of BW	Probiotics: *Lactobacillus reuteri*	Orally gavaged, for 8 weeks	Normal chow diet	BW↑,AST↓,ALT↓,TC↓,TG↓,Endotoxin↓ and TNF-α↓
Yi et al. (46)	Male C57BL/6N mice 8-week-old	56	Via stomach injection ethanol	Probiotics: Dried probiotic tablets containing Bifidobacterium infantis, B. animalis, and *Lactobacillus acidophilus*	Orally gavaged, for 10 days	Isocaloric maltose-dextrin	MDA↓,GSH↑,SOD↑
Zheng et al. (48)	Male C57BL/6N mice 7-week-old	42	Orally treated with alcohol for 28 days	Probiotics: *Lactobacillus plantarum*; *Lactobacillus fermentum*; *Lactobacillus reuteri*	Orally gavaged, for 28 days	Normal chow diet	AST↓,ALT↓,TG↓,GSH↑,IL-6↓ and TNF-α↓
Fan et al. (49)	Male C57BL/6N mice 8-week-old	40	Orally gavaged 5% (v/v) ethanol	Prebiotics: Polysaccharides from crassostrea gigas (RPS); Polysaccharides from steamed oyster (SPS)	Orally gavaged, for 28 days	Lieber-DeCarli diet	AST↓,ALT↓,Endotoxin↓,TNF-α↓,IL-1β↓and microbial transform
Jiang et al. (52)	Youngmale Wistar rats	38	Orally gavaged 6% (vol/vol) Lieber-DeCarli liquid diet	Probiotics: *Lactobacillus acidophilus*; *Lactobacillus paracasei*; *Lactobacillus delbrueckii*	Orally gavaged, for 25 days	Isocaloric maltose-dextrin	ALT↓,AST↓,ALP↓,TC↓,TG↓,TNF-α↓,IL-1β↓,TLR-4↓
Lu and Wang (4)	Male C57BL/6N mice 7–8-week-old	40	Orally gavaged 5% (v/v) ethanol	Probiotics: *Lactobacillus acidophilus*	Orally gavaged, for 14 days	Isocaloric maltose-dextrin	ALT↓,AST↓,SOD↑,MDA↓,GSH↑,IL-1β↓,TNF-α↓,Endotoxin↓,TG↓and microbial transform
Gan et al. (50)	Adult male Wistar rats	60	Orally gavaged high dietary iron (1,500 mg/kg) and 56% v/v alcohol	Probiotics: *Lactobacillus casei*	Orally gavaged, for 12 weeks	Normal saline	ALT↓,GGT↓,TG↓,Endotoxin↓and TNF-α↓
Nam et al. (42)	C57BL/6 mice (8–10 weeks)	28	Orally gavaged 5% (v/v) ethanol	Probiotics: *Pediococcus pentosaceus*	Orally gavaged, for 11 days	Isocaloric maltose-dextrin	ALT↓,AST↓,TG↓,IL-6↓,TNF-α↓and microbial transform
You et al. (47)	Male Kunming mice	40	Orally gavaged 56,ethanol	Probiotics: 1. *Lactobacillus plantarum* HFY09; 2. *Lactobacillus delbruechill* subsp. Bulgaricus	Orally gavaged, for 7 days	Normal saline	AST↓,ALT↓,TG↓,IL-6↓,TNF-α↓,IL-1β↓,SOD↑,MDA↓,GSH↑,TC↓ and TG↓
Li et al. (53)	Male C57BL/6N mice 4–6-week-old	30	Orally treated with alcohol	Probiotics: *Bacillus subtilis*	Orally, for 16 days	Isocaloric maltose-dextrin	ALT↓,AST↓,TG↓,MDA↓,Endotoxin↓,IL-6↓

↑ and ↓ represent increased or decreased outcome indicators in the treatment group compared with control group, respectively. ALP, alanine aminotransferase; AST, aspartate transaminase; ALP, alkaline phosphatase; GGT, gamma-glutamyl transferase; TNA-α, tumor necrosis factor-α; IL-6, interleukin-6; IL-1β, interleukin –1β; SOD, superoxide dismutase; MDA, malondialdehyde; GSH, glutathione; TG, triglyceride; TC, total cholesterol; TB, total bilirubin; CFU, colony-forming unit.

**TABLE 2:** Characteristics of included studies investigating the effects of probiotics and prebiotics on human ALD.

References	Country	Sample size	Age	Intervention of experimental group	Route and duration	Comparison	Outcome indicators
Grander et al. (10)	Russian	66	18 years or older	*Lactobacilli* and *Bifidobacteria*	12 weeks	Vitamin B1 and B6	ALT↓, AST↓, and TB↓
Peters et al. (17)	European	20	18 and 75 years old	*Lactobacillus casei* Shirota	4 weeks	Placebo	TB↓, ALT↓, and TNF-a↓
Zhao et al. (24)	USA	117	52.7 ± 11.3 (year)	*Lactobacillus subtilis* or *Streptococcus faecium*	7 days	Placebo	AST↓, ALT↓, TB↓, and TNF-α↓
Hsieh et al. (51)	China	158	30–65 years old	*Lactobacillus casei* strain	60 days	Placebo	ALT↓, AST↓, and TNF-a↓

Two reviewers independently assessed method quality using the SYRCLE tool. Blinding caused most biases. The items were judged as low, unclear, or high risks. In most studies, blinding and allocation bias were unclear because no specific details of relevant information were provided. All studies were rated as having a low risk of reporting bias. Overall, the studies had similar high-quality evaluations with a low risk of bias (Supplementary Tables 1, 2). Differences in the quality evaluation process were addressed through discussion.

### Effect of microbial agents on lipid control

**FIGURE 2:**
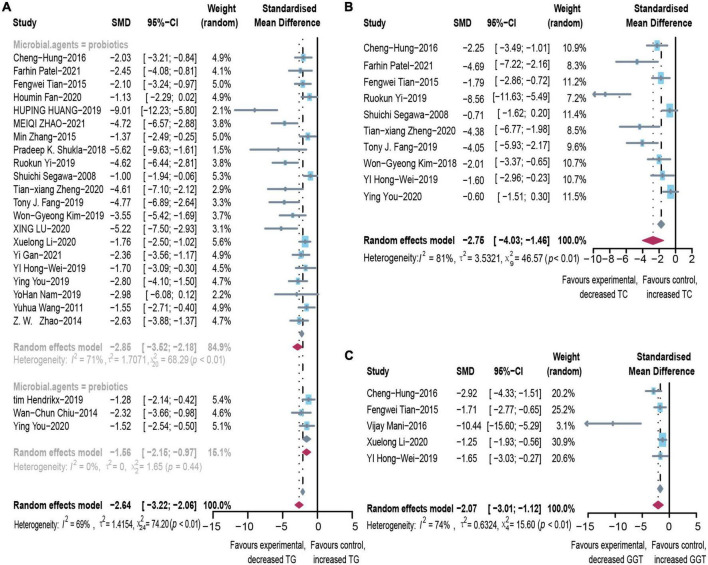
Effectiveness of microbial agents on lipid index. **(A)** The effect of microbial agents on TG, **(B)** TC. **(C)** The effect of microbial agents on GGT. SMD, Standardized mean difference; CI, Confidence interval.

**FIGURE 3:**
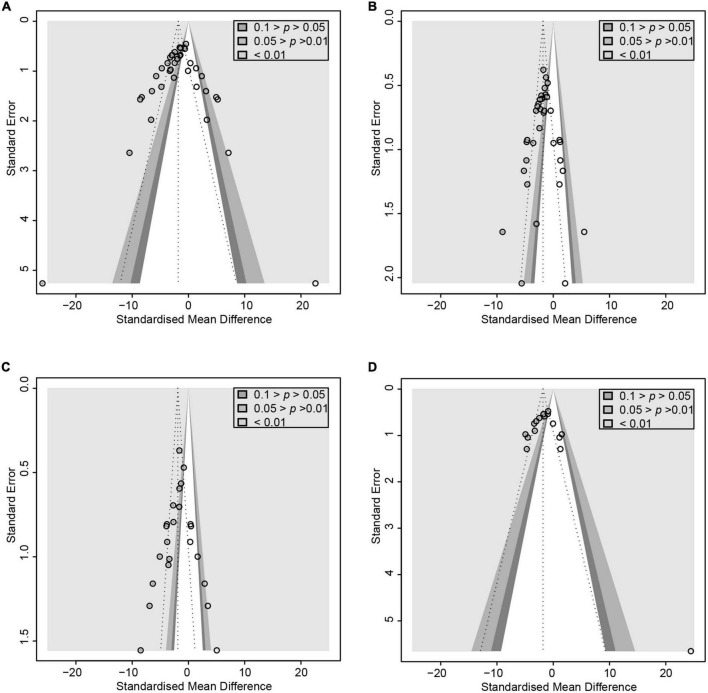
Contour-enhanced funnel plot with trim-and-fill method. **(A)** ALT. **(B)** TG **(C)** TNF-α **(D)** Endotoxin. If the missing studies were in the non-significant area, the asymmetry was due to publication bias. Otherwise, the observed asymmetry could be attributed to factors other than publication bias.

Microbial therapy has a moderating effect on hyperlipemia, as indicated by a more dampened level of TC (SMD = -2.75, 95% CI –4.03 to –1.46, *I*^2^ = 81%) and TG (SMD = –2.64, 95% CI –3.22 to –2.06, *I*^2^ = 69%) (Figure 2). Subgroup analysis showed that biopsy tissue and mouse species are responsible for high TG heterogeneity (Supplementary Table 3). The contour-enhanced funnel plot with the trim-and-fill method indicates that publication bias was not the leading cause of asymmetry (Figure 3). Sensitivity analysis confirmed the robustness of the study. There were no clinical studies. About the effect of microbial agents on lipid control.

### Effect of microbial agents on liver biochemical indicators

**FIGURE 4:**
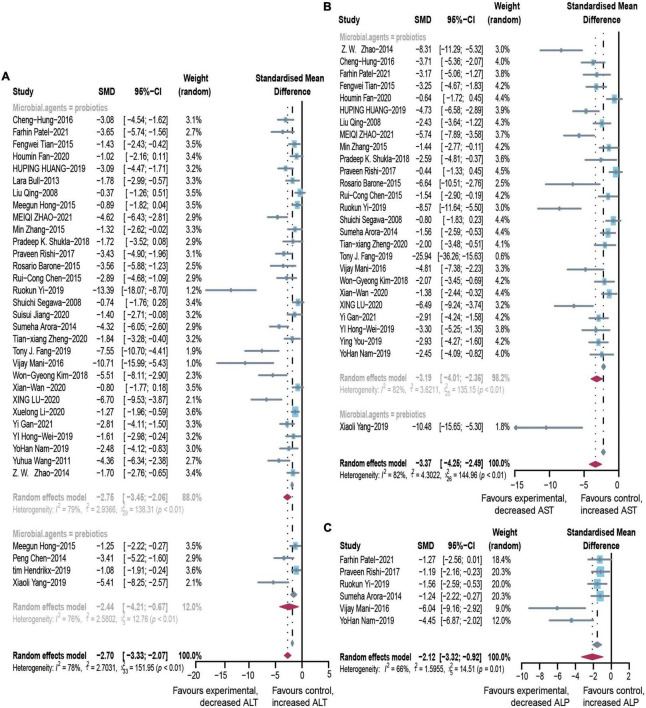
Effectiveness of microbial agents on liver biochemical index. **(A)** The effect of microbial agents on ALT, **(B)** AST, and **(C)** ALP. SMD, Standardized mean difference; CI, Confidence interval.

After pooled analysis of the data, there were significant differences in ALT (SMD: –2.70, 95% CI: –3.33 to –2.07, I^2^ = 78%), AST (SMD: –3.37, 95% CI: –4.25 to –2.49, *I*^2^ = 82%) and alkaline phosphatase (SMD: –2.12, 95% CI: –3.32 to –0.92, *I*^2^ = 66%), between the experimental and control groups (Figure 4). In addition, GGT (SMD: –1.8, 95% CI: –2.39 to –1.24, *I*^2^ = 68%), which is more specific to ALD, has also been addressed (Figure 2). Because of significant heterogeneity, the reasons for these differences were investigated by conducting subgroup analyses (Supplementary Table 4). The heterogeneity of ALT was slightly altered after considering probiotics and prebiotics separately; however, the heterogeneity changed more significantly when the variables were controlled for the animal model, flora type, and feeding pattern. This finding suggested that the more significant heterogeneity may be due to these factors. There was an inconspicuous asymmetry in the contour-enhanced funnel plot. The trim-and-fill method demonstrated that the asymmetry was caused by factors other than publication bias (Figure 3).

There were many reports investigating the role of probiotics in experimental ALD. Clinical studies were rare, but we have conducted a careful analysis, according to the clinical study data we have obtained: ALT (SMD: –0.95, 95% CI: –0.40 to –1.1, *I*^2^ = 69%), AST (SMD: –1.4, 95% CI: –3.2 to –0.4, *I*^2^ = 97%), and GGT (SMD: –0.63, 95% CI: –1.07 to –0.20, *I*^2^ = 70%) decreased significantly compared to the control group (Supplementary Figure 2).

### Effect of microbial agents on inflammation mediators

**FIGURE 5:**
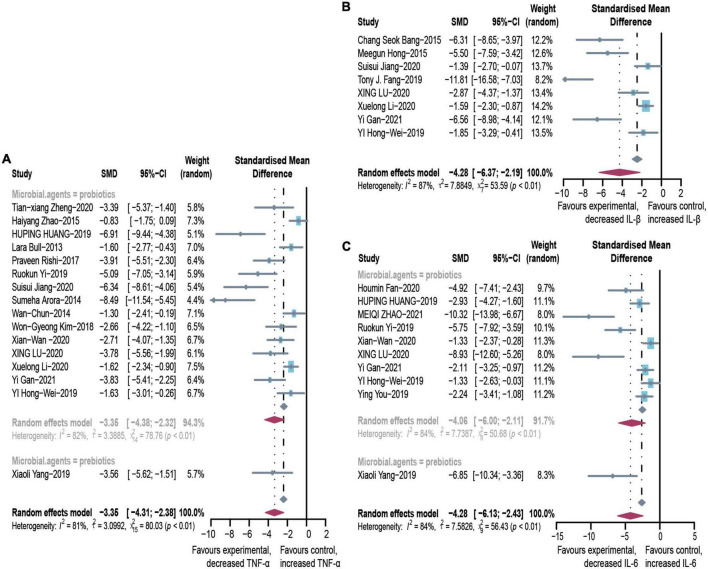
Effectiveness of microbial agents on inflammatory cytokines. **(A)** The effect of microbial agents on TNF-α. **(B)** The effect of microbial agents on IL-1β. **(C)** The effect of microbial agents on IL-6. SMD, Standardized mean difference; CI, Confidence interval.

In animal studies, TNF-α, IL-6, and IL-1β were used to assess inflammatory infiltration due to ALD (Figure 5). There were lower levels of TNF-α (SMD: –3.35, 95% CI: –4.31 to –2.38, *I*^2^ = 81%), IL-6 (SMD: –4.28, 95% CI: –6.13 to –2.43, *I*^2^ = 84%), and IL-1β (SMD: –4.28, 95% CI: –6.37 to –2.19, *I*^2^ = 87%), with evident heterogeneity. The analysis of subgroups was based on three items including animal models, tissues, and routes. The heterogeneity changed markedly among the animal models, suggesting that differences in animal species may be responsible for heterogeneity (Supplementary Table 5). The pooled analysis of TNF-α in serum and liver suggests that different tissues might not be the source of the heterogeneity. Egger’s test indicated publication bias and profile-enhanced funnel plots (drawn using the trim-and-fill method; Figure 3) showed that publication bias was not the leading cause of asymmetry. Sensitivity analysis revealed that no studies interfered significantly with the meta-analysis, implying good stability.

Due to the paucity of clinical literature on indicators of inflammation, we only performed a pooled analysis of TNF-α (SMD: –1.7, 95% CI: –4.39 to 0.9, *I*^2^ = 89%). This finding suggests that probiotics moderate inflammation (Supplementary Figure 2).

### Effect of microbial agents on floral translocation and endotoxin

A comprehensive study of intestinal flora translocation and endotoxin was conducted to evaluate the changes of each in patients with ALD. Intestinal flora underwent dramatic changes in response to alcohol (Supplementary Figure 1), with most flora showing an upward trend, including *Lactobacillus* (SMD: 4.40, 95% CI: 0.97–7.84, *I*^2^ = 85%), *Bifidobacteria* (SMD: 3.84, 95% CI: 0.22–7.45, *I*^2^ = 88%), and *Bacteroidetes* (SMD: 2.51, 95% CI: 0.29–4.72, *I*^2^ = 80%). *Proteobacteria* proliferated even more (SMD: –4.18, 95% CI: –6.60 to –1.77, *I*^2^ = 86%). Interestingly, most proliferating bacteria were beneficial to the intestinal tract (e.g., *Lactobacillus*); however, this finding could be because the microbial preparations administered in the animal models were *Lactobacillus*. We also explored the variation in endotoxin (SMD: –2.70, 95% CI: –3.52 to –1.88, *I*^2^ = 79%). Contour-enhanced funnel plots using the trim-and-fill method showed that publication bias was not the primary cause of asymmetry (Figure 3). The robustness of the results was demonstrated by sensitivity analysis. There were no clinical studies investigating floral translocation and endotoxin.

### Effect of microbial agents on oxidative stress

**FIGURE 6:**
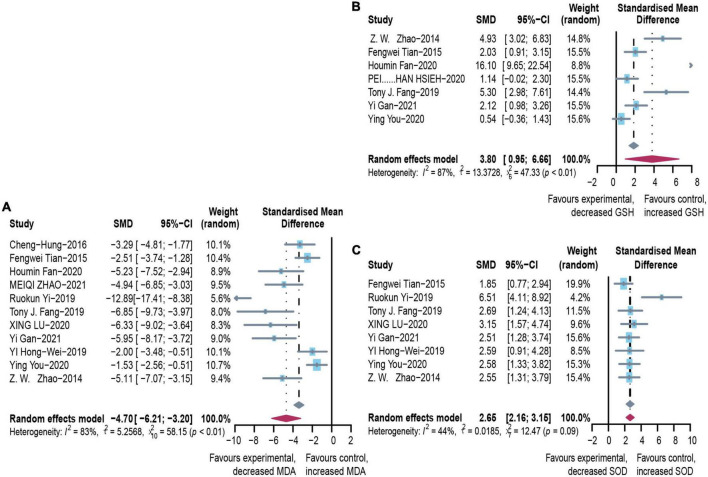
Effectiveness of microbial agents on oxidative stress. **(A)** The effect of microbial agents on MDA to evaluate free radical mediated lipid peroxidation injury. **(B,C)** The effect of microbial agents on GSH and SOD to evaluate antioxidant status of tissues. SMD, Standardized mean difference; CI, Confidence interval.

To evaluate the free radical-mediated lipid peroxidation damage and the antioxidant status of tissues, we measured levels of glutathione (GSH), SOD, and MDA (Figure 6). Microbial agent treatment contributed to increased levels of SOD (SMD: 2.65, 95% CI: 2.16–3.15, *I*^2^ = 44%) and GSH (SMD: 3.80, 95% CI: 0.95–6.66, *I*^2^ = 87%), while there was a significant decrease in MDA (SMD: –4.70, 95% CI: –6.21 to –3.20, *I*^2^ = 83%) with considerable heterogeneity. We performed a subgroup analysis of MDA according to mice models and feeding practices and obtained no meaningful results. Asymmetry was present in the contour-enhanced funnel plot (Figure 3), demonstrating that publication bias was not the leading cause of asymmetry. There were no clinical studies.

## Discussion

We identified convincing evidence for the use of microbial treatment of ALD through careful analysis of clinical trials and animal studies. To our knowledge, this is the first meta-analysis of such therapies. Although alcohol is primarily metabolized in the liver, alcohol consumption causes ecological dysregulation of bacteria in the intestine, damage to the intestinal mucosa, and increased intestinal permeability, resulting in increased transport of bacteria and their products (e.g., endotoxins) into the portal circulation. The increased inflammatory cytokine levels during liver injury reach the intestine through the circulation to damage the intestinal mucosal barrier. This phenomenon disrupts the balance of the intestinal flora, creating a vicious cycle (57). Once the dynamic balance is disturbed under the onslaught of pathogenic factors, intestinal and hepatic dysfunction is triggered. Fortunately, probiotics and prebiotics can effectively maintain intestinal homeostasis (58).

*Lactobacillus* and *Bifidobacterium* are the most predominant antimicrobial genera among probiotics. Most studies chose probiotics containing *Lactobacillus*, *Bifidobacterium*, or a mixture of the two. These beneficial bacteria compete with pathogenic bacteria for binding sites in the intestinal epithelium, and they effectively reduce the pathogenic microorganisms by releasing antibacterial substances such as lactic acid and hydrogen peroxide (59). We found that, after the probiotic intervention, the dysbiosis in ALD mice improved with increased abundance of *Bifidobacterium*, decreased *Proteobacteria*, and a corresponding decrease in the rate of intestinal infections was also observed. *Bifidobacteria* and *Lactobacilli* are thought to promote mucosal immunity through the intestinal microbiota (60). Other potentially beneficial flora such as *Lachnospiraceae* might promote intestinal mucosal integrity through the metabolite butyrate, a short-chain fatty acid (61). Our findings demonstrated that the intestinal epithelial barrier (IEB) was more consolidated than the model groups, suggesting that probiotics and prebiotics can reinforce each other and work together to maintain intestinal immunity (62).

In addition to active modification of the intestinal flora, studies reported that probiotics protect the intestine and liver from alcohol stimulation by regulating the synthesis, catabolism and lipid transport, mitigating oxidative stress, and reinforcing the IEB (63). Sterol regulatory element binding proteins (SREBPs) are transcriptional mediators of lipid homeostasis that are upregulated in response to alcohol abuse, raising hepatic steatosis and plasma TG levels. SREBP-1c is the primary regulator of hepatic fatty acid and TG synthesis, and SREBP-2 regulates cholesterol synthesis (64). Alcohol reduces the expression of PPAR-α and MTP, critical participants in the transfer of TG and TC in the liver, leading to increased lipid accumulation (65). Probiotics inhibit weight gain, epiphyseal adipose tissue expansion, and partially reverse fructose (FRD)-induced adipocyte hypertrophy (66). Probiotics prevent the elevation of plasma triglycerides, leptin, and hepatic TG levels (67). Prebiotic fermentation products increased the production of hepatic mucin and modulated the action of hepatic lipogenic enzymes. Our findings suggest a significant decrease in TG and TC in the liver and serum compared to the model group (68).

Alcohol-induced inflammatory reactions should also be considered in the liver and throughout the body. The oxidative pathway of alcohol metabolism mediated by ethanol dehydrogenase and acetaldehyde dehydrogenase produces large amounts of acetaldehyde, which is thought to be the primary mediator of alcohol toxicity in the liver (69). Excess acetaldehyde displaces the intestinal flora and damages the intestinal mucosal barrier (70). Intestinal bacteria-derived endotoxins function through pattern recognition receptors such as TLRs, expressed in hepatic cells such as Kupffer cells. Lipopolysaccharide-induced inflammation boosts inflammatory cytokines, including TNF-α and IL-1β, which stimulate Stimulates NF-κB activation through the MAPK (mitogen-activated protein kinase) pathway, and the activated NF-κB enters the nucleus, forming a cytokine-NF-κB loop and causing a series of inflammatory responses in the cells (71). Consistent with these findings, we found that human trials and animal studies showed a considerable increase in inflammatory parameters in ALD model compared to the control group.

In the inflammatory cascade, unsaturated fatty acids are driven by reactive oxygen species (ROS) to produce lipid peroxidases, which trigger fatty acid side chain reactions. Oxidative metabolites of ethanol, such as acetaldehyde and ROS, play essential roles in the clinical and pathological spectra ALD’s. At the same time, excess acetaldehyde entering the bloodstream is converted to superoxide by p-xanthine oxidase, producing MDA, the end product of free radical-mediated lipid peroxidation, which is used as a marker of oxidative stress (72). The antioxidants SOD and GSH reflect antioxidant levels. Ethanol-induced oxidative stress damage was confirmed by the decreased levels of SOD and GSH and the high content of MDA in alcohol-fed mice (73). The presence of microorganisms not only ameliorates oxidative stress by suppressing ROS and significantly reducing cytokine levels by inhibiting TLR-mediated endotoxins (21). Our findings showed that probiotics or prebiotics could reduce MDA levels by inhibiting the inflammatory response and the oxidative effect of alcohol. Meanwhile, there were increased concentrations of the antioxidants SOD and GSH.

The toxic effects of alcohol on the liver are mediated by interfering with lipid metabolism, disrupting the mucosal barrier, enhancing the inflammatory response and promoting oxidative stress. In contrast, microbial treatment can lead to significant changes in liver-specific biological enzymes. In clinical trials, we could observe a greater decrease in AST, ALT, and GGT compared to the control group. Interestingly, In patients with alcoholic liver disease, elevations in AST were more pronounced than in ALT and serum AST concentrations are usually more than twice as high as ALT because alcohol induces mitochondrial dysfunction through activation of the CYP2E1 enzyme and because of the massive release of AST from the mitochondrial matrix (74). And in animal experiments we obtained the same results, which fully illustrates the incredible effect of microbial treatment for ALD.

Our meta-analysis of 41 studies showed that microbial agents could help to treat ALD; nevertheless, there were still some limitations. First, due to the exploratory nature of this study, heterogeneity was inevitable when combining specific indicators, even when using random effects models and subgroup analysis. Nevertheless, sensitivity analysis supported the robustness of our results. Asymmetry appeared in the funnel plot and Egger’s test, indicating publication bias. Contour-enhanced funnel plots using the trim-and-fill method demonstrated that heterogeneity was the primary cause of asymmetry. In addition, although the number of clinical studies is limited and the data used for statistical analysis is small, we have conducted a careful analysis to summaries while hoping that more clinical studies will be available to support our conclusion. In this light, the conclusions we drew from 41 articles remain valid.

## Conclusion

Prebiotics and probiotics exert hepatoprotective effects by regulating intestinal flora, maintaining the integrity of the intestinal mucosa, reducing the entry of endotoxins released by pathogenic microorganisms into the portal system, and inhibiting oxidative stress as well as pro-inflammatory factors. Our study provides new insights into the management of ALD. Nevertheless, clinical studies are still needed to translate microbial therapy into practical clinical applications.

## Data availability statement

The original contributions presented in this study are included in the article/Supplementary material, further inquiries can be directed to the corresponding author/s.

## Author contributions

ZX conceived the idea and designed the study strategy. QW, JS, and MZ conducted reference search. QW and CX summarized the data. QW, GR, YX, and OY drafted the manuscript. QW conducted data acquisition and statistical analyses. FW provided critical revisions of the manuscript for important intellectual content, administrative and funding support, and supervision. All authors contributed to the article and approved the submitted version.
