# Supplementary material for: Microbial treatment of alcoholic liver disease: A systematic review and meta-analysis
Source: Front Nutr. 2022 Nov 21;9:1054265. doi: 10.3389/fnut.2022.1054265 (PMC9719948; doi:10.3389/fnut.2022.1054265)
Supplement: Supplementary file 1 [file Data_Sheet_1.docx]

Supplementary Materia

**Microbial treatment of alcoholic liver disease: a systematic review and meta-analysis**

**Qinjian Wang^1^**^†^**, Jiangmin Shi^1^**^†^**, Min Zhao^1^**^†^**, Gaoyi Ruan^1^, Zebin Dai^1^, Yilang Xue^1^, Yaoxuan Chen^1^, Dibang Shi^1^, Changlong Xu^1^, Ouyue Yu^2^, Fangyan Wang^2*^, and Zhanxiong Xue^1*^**

^1^ Department of Gastroenterology, The Second Affiliated Hospital and Yuying Children’s Hospital of Wenzhou Medical University, Wenzhou, Zhejiang Province, China;

^2^ Department of Pathophysiology, School of Basic Medicine Science, Wenzhou Medical

University, Wenzhou, China.

† These authors have contributed equally to this work.

***Co-corresponding authors:**

Dr.Fangyan Wang

Department of Pathophysiology, School of Basic Medicine Science, Wenzhou Medical

University, Wenzhou, China

Wenzhou 325000, Zhejiang Province, China

Phone: +86(0577)88002715; Fax: +86(0577)88002715

E-mail: fangyan_wang@wmu.edu.cn

***Corresponding authors:**

Dr.Zhanxiong Xue

The Second Affiliated Hospital and Yuying Children’s Hospital of Wenzhou Medical University

Wenzhou 325000, Zhejiang Province, China

Phone: +86-577-86687632; Fax: +86-577-86689848

E-mail: xuezhanxiong@126.com

Keywords: alcoholic liver disease, microbial agents, probiotics, prebiotics, gut-liver axis, meta-analysis

# Supplementary Figures and Tables

## Supplementary Tables

**Supplementary Table 1. Risk of bias summary** Judgements about each risk of bias item for each included animal study.

|  | | **Selection bias** | | | **Performance bias** | | **Detection bias** | | **Attrition bias** | **Reporting bias** | **Other** |
| --- | --- | --- | --- | --- | --- | --- | --- | --- | --- | --- | --- |
| **Study** | **Year** | **Sequence  generation** | **Baseline  characteristics** | **Allocation  concealment** | **Random housing** | **Blinding** | **Random outcome  assessment** | **Blinding** | **Incomplete outcome  data** | **Selective outcome  reporting** | **Other sources of bias** |
| **L. Qing et al.** | 2008 | unclear | low risk | unclear | low risk | high risk | unclear | unclear | low risk | low risk | unclear |
| **Shuichi Segawa et al.** | 2008 | low risk | low risk | unclear | low risk | high risk | unclear | unclear | low risk | low risk | low risk |
| **Yuhua Wang et al.** | 2011 | unclear | low risk | unclear | unclear | high risk | low risk | unclear | low risk | low risk | low risk |
| **Lara Bull-Otterson et al.** | 2013 | unclear | low risk | unclear | low risk | high risk | low risk | unclear | unclear | low risk | low risk |
| **Sumeha Arora et al.** | 2014 | low risk | low risk | unclear | low risk | high risk | low risk | unclear | low risk | low risk | unclear |
| **Z. W. Zhao et al.** | 2014 | low risk | low risk | unclear | low risk | high risk | low risk | unclear | low risk | low risk | low risk |
| **Peng Chen et al.** | 2014 | unclear | low risk | unclear | low risk | high risk | low risk | unclear | low risk | low risk | low risk |
| **Wan-Chun Chiu et al.** | 2015 | low risk | low risk | unclear | low risk | high risk | unclear | unclear | low risk | low risk | unclear |
| **Meegun Hong et al.** | 2015 | unclear | unclear | unclear | low risk | high risk | low risk | unclear | low risk | low risk | low risk |
| **Haiyang Zhao et al.** | 2015 | low risk | low risk | unclear | low risk | high risk | low risk | unclear | low risk | low risk | unclear |
| **Fengwei Tian et al.** | 2015 | low risk | low risk | unclear | low risk | high risk | low risk | unclear | low risk | low risk | unclear |
| **Min Zhang et al.** | 2015 | unclear | low risk | unclear | low risk | high risk | low risk | unclear | unclear | low risk | low risk |
| **Rosario Barone et al.** | 2016 | unclear | low risk | unclear | unclear | high risk | low risk | unclear | low risk | low risk | low risk |
| **Rui-Cong Chen et al.** | 2016 | low risk | low risk | unclear | unclear | high risk | unclear | unclear | low risk | low risk | low risk |
| **Cheng-Hung Chuang et al.** | 2016 | low risk | low risk | unclear | low risk | high risk | low risk | unclear | unclear | low risk | unclear |
| **Vijay Mani et al.** | 2016 | low risk | low risk | unclear | unclear | high risk | low risk | unclear | low risk | low risk | low risk |
| **Praveen Rishi et al.** | 2016 | low risk | low risk | unclear | low risk | high risk | low risk | unclear | low risk | low risk | unclear |
| **Won-Gyeong Kim et al.** | 2018 | low risk | low risk | unclear | low risk | high risk | low risk | unclear | unclear | low risk | unclear |
| **Pradeep K. Shukla et al.** | 2018 | low risk | low risk | unclear | unclear | high risk | unclear | unclear | low risk | low risk | low risk |
| **Tony J. Fang et al.** | 2019 | low risk | low risk | unclear | unclear | high risk | low risk | unclear | low risk | low risk | unclear |
| **Tim Hendrikx et al.** | 2018 | low risk | low risk | unclear | low risk | high risk | unclear | unclear | low risk | low risk | low risk |
| **HUPING HUANG et al.** | 2019 | low risk | low risk | unclear | low risk | high risk | unclear | unclear | low risk | low risk | low risk |
| **YoHan Nam et al.** | 2019 | low risk | low risk | unclear | low risk | high risk | low risk | unclear | low risk | low risk | unclear |
| **Xiaoli Yang et al.** | 2019 | unclear | low risk | unclear | low risk | high risk | low risk | unclear | unclear | low risk | unclear |
| **Ruokun Yi et al.** | 2019 | unclear | low risk | unclear | low risk | high risk | low risk | unclear | low risk | low risk | unclear |
| **YI Hong-Wei et al.** | 2020 | unclear | low risk | unclear | low risk | high risk | low risk | unclear | low risk | low risk | low risk |
| **Ying You et al.** | 2020 | low risk | low risk | unclear | low risk | high risk | unclear | unclear | low risk | low risk | low risk |
| **Tian-xiang Zheng et al.** | 2020 | unclear | low risk | unclear | low risk | high risk | low risk | unclear | low risk | low risk | low risk |
| **Houmin Fan et al.** | 2021 | low risk | low risk | unclear | unclear | high risk | low risk | unclear | low risk | low risk | low risk |
| **PEI‑SHAN HSIEH et al.** | 2020 | unclear | low risk | unclear | low risk | high risk | low risk | unclear | low risk | low risk | unclear |
| **Suisui Jiang et al.** | 2021 | unclear | low risk | unclear | low risk | high risk | low risk | unclear | unclear | low risk | low risk |
| **Farhin Patel et al.** | 2021 | unclear | low risk | unclear | unclear | high risk | low risk | unclear | low risk | low risk | unclear |
| **XING LU et al..** | 2021 | low risk | low risk | unclear | low risk | high risk | low risk | unclear | low risk | low risk | low risk |
| **Xuelong Li et al.** | 2021 | unclear | low risk | unclear | low risk | high risk | unclear | unclear | unclear | low risk | unclear |
| **Xian-Wan Jiang et al.** | 2021 | unclear | low risk | unclear | low risk | high risk | unclear | unclear | unclear | low risk | low risk |
| **Yi Gan et al.** | 2021 | low risk | low risk | unclear | low risk | high risk | low risk | unclear | unclear | low risk | low risk |
| **MEIQI ZHAO et al.** | 2021 | low risk | low risk | unclear | low risk | high risk | low risk | unclear | low risk | low risk | low risk |

**Supplementary Table 2. Risk of bias summary** Judgements about each risk of bias item for each included clinical study.

|  | | **Selection bias** | | | **Performance bias** | | **Detection bias** | | **Attrition bias** | **Reporting bias** | **Other** |
| --- | --- | --- | --- | --- | --- | --- | --- | --- | --- | --- | --- |
| **Study** | **Year** | **Sequence  generation** | **Baseline  characteristics** | **Allocation  concealment** | **Random housing** | **Blinding** | **Random outcome  assessment** | **Blinding** | **Incomplete outcome  data** | **Selective outcome  reporting** | **Other sources of bias** |
| **Irina A. Kirpich et al.** | 2008 | low risk | unclear | unclear | low risk | unclear | unclear | unclear | low risk | low risk | unclear |
| **Vanessa Stadlbauer et al.** | 2008 | unclear | unclear | unclear | unclear | unclear | unclear | unclear | low risk | low risk | unclear |
| **Sang Hak Han et al.** | 2015 | low risk | low risk | unclear | low risk | unclear | low risk | unclear | low risk | low risk | unclear |
| **Xuelong Li et al.** | 2020 | low risk | unclear | unclear | low risk | low risk | unclear | unclear | low risk | low risk | unclear |

**Supplementary Table 3. Subgroup analysis of TG in preclinical studies**

|  | **No.of studies** | **SMD (95%)** | **I^2^(%)** | **p-value** |
| --- | --- | --- | --- | --- |
| **Microbial agents** | | | | |
| Probiotics | 30 | -3.00 (-3.64, -2.36) | 74 | <0.001 |
| Prebiotics | 3 | -1.56 (-2.15, -0.97) | 0 | 0.44 |
| **Animal model** | | | | |
| C57BL/6 | 18 | -1.99 (-2.30, -1.68) | 81 | <0.001 |
| Wistar | 3 | -2.09 (-2.92, -1.26) | 0 | 0.67 |
| Kunming | 7 | -2.73 (-3.25, -2.22） | 76 | <0.001 |
| Other | 5 | -2.26 (-2.82, -1.71) | 10 | 0.35 |
| **Tissue** | | | | |
| Liver | 16 | -2.41(-3.05, -1.77) | 66 | <0.001 |
| serum | 17 | -3,14 (-4.42, -2.27) | 74 | <0.001 |

**Supplementary Table 4. Subgroup analysis of ALT in preclinical studies**

|  | **No.of studies** | **SMD (95%)** | **I^2^(%)** | **p-value** |
| --- | --- | --- | --- | --- |
| **Microbial agents** | | | | |
| Probiotics | 42 | -1.94 (-2.16, -1.71) | 80 | <0.001 |
| Prebiotics | 4 | -2.20 (-2.92, -1.48) | 75 | <0.001 |
| **Animal model** | | | | |
| C57BL/6 | 16 | -1.62 (-1.92, -1.33) | 76 | <0.001 |
| Wistar | 5 | -2.00 (-2.73, -1.28) | 52 | 0.03 |
| Sprague-Dawley | 3 | -1.92 (-2.73, -1.12) | 89 | <0.001 |
| Kunming | 3 | -4.60 (-6.56, -2.64） | 83 | 0.02 |
| Other | 2 | -1.20 [-2.10, -0.30] | 78 | 0.03 |
| **Bacterial strains** | | | | |
| Lactobacillus | 29 | -1.97 (-2.20, -1.73) | 76 | <0.001 |
| Other | 5 | -1.79 (-2.41, -1.18) | 90 | <0.001 |

**Supplementary Table 5. Subgroup analysis of TNF-α in preclinical studies**

|  | **No. of studies** | **SMD (95%)** | **I^2^(%)** | **p-value** |
| --- | --- | --- | --- | --- |
| **Tissue** | | | | |
| Liver | 12 | -4.09 (-5.70, -2.48) | 86 | <0.001 |
| Serum | 13 | -3.82 (-4.86, -2.78) | 79 | <0.001 |
| **Animal model** | | | | |
| C57BL/6 | 7 | -2.81 (-3.42, -2.21) | 21 | <0.001 |
| Wistar | 6 | -2.45 (-2.98, -1.93) | 89 | <0.001 |
| Sprague-Dawley | 3 | -4.94 (-6.11, -3.77) | 79 | <0.001 |
| Kunming | 5 | -3.33 (-4.03, -2.64） | 81 | 0.004 |
| Other | 2 | -1.20 [-2.10, -0.30] | 78 | 0.03 |

## Supplementary Figure

**Supplementary Figure1
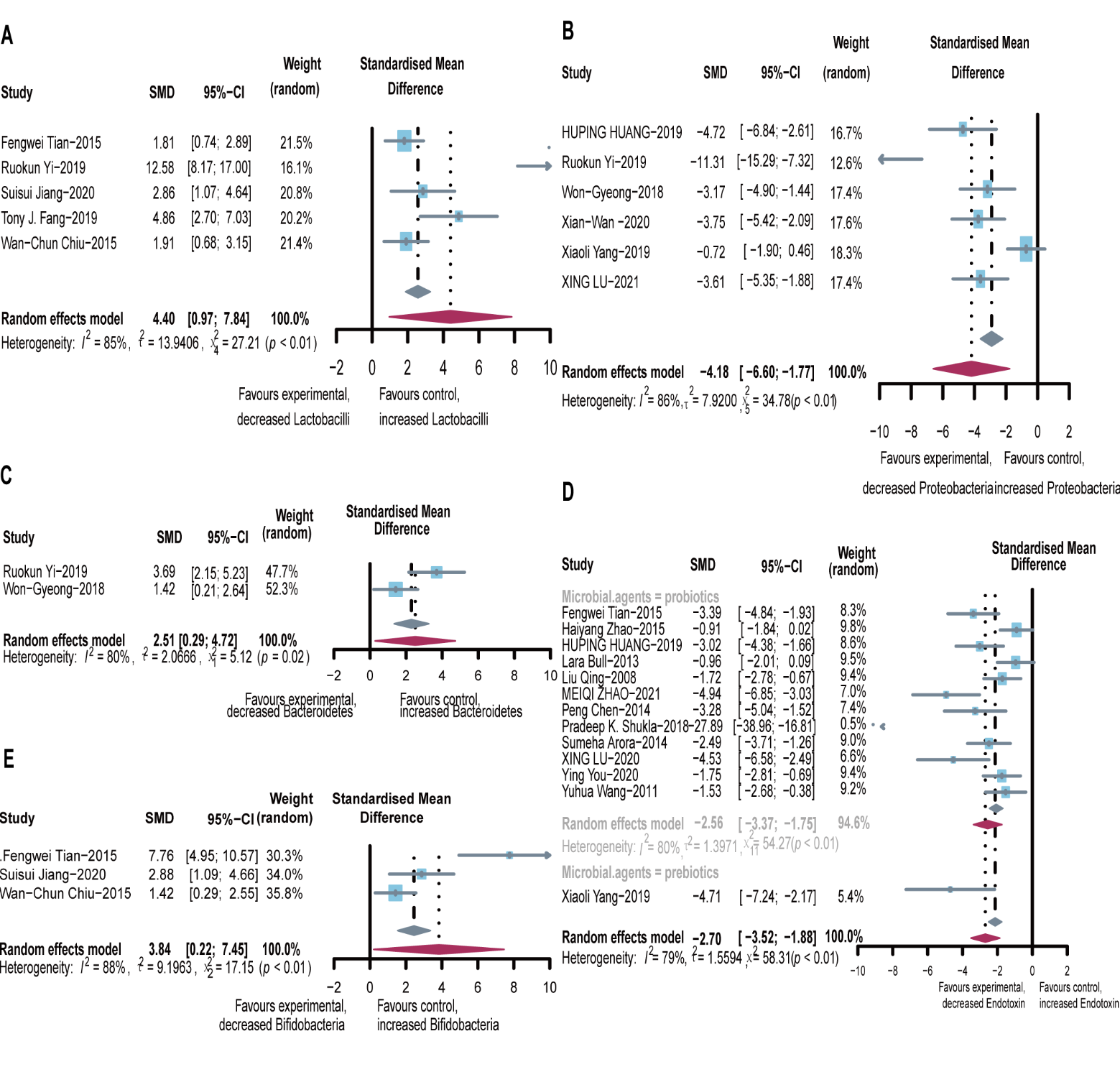
**

**Supplementary Figure 2 Effectiveness of microbial agents on clinical study. (A)** The effect of microbial agents on ALT, (**B**) AST, (**C**) GGT, (**D**) TNA-α SMD = Standardized mean difference; CI = Confidence interval

**
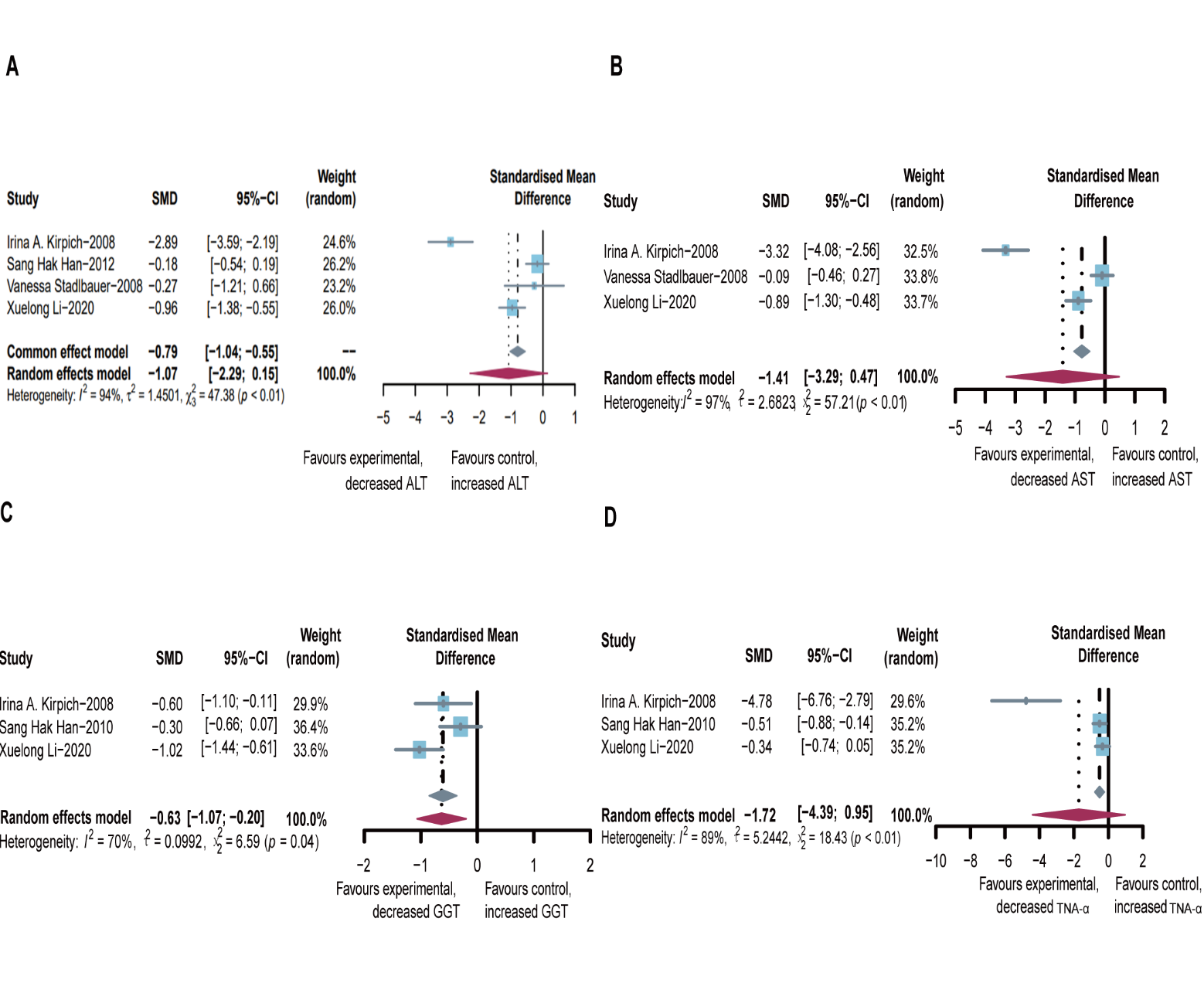
**
